# Clinical, biochemical, and genetic analysis of 28 Chinese patients with holocarboxylase synthetase deficiency

**DOI:** 10.1186/s13023-023-02656-y

**Published:** 2023-03-08

**Authors:** Shiying Ling, Wenjuan Qiu, Huiwen Zhang, Lili Liang, Deyun Lu, Ting Chen, Xia Zhan, Yu Wang, Xuefan Gu, Lianshu Han

**Affiliations:** Department of Pediatric Endocrinology and Genetic Metabolism, Shanghai Institute for Pediatric Research, Xinhua Hospital, Shanghai Jiaotong University School of Medicine, Shanghai, 200092 China

**Keywords:** Holocarboxylase synthetase deficiency, *HLCS*, Newborn screening, Tandem mass spectrometry

## Abstract

**Background:**

This study aimed to describe the clinical, biochemical, and molecular characteristics of Chinese patients with holocarboxylase synthetase (HLCS) deficiency, and to investigate the mutation spectrum of HCLS deficiency as well as their potential correlation with phenotype.

**Methods:**

A total of 28 patients with HLCS deficiency were enrolled between 2006 and 2021. Clinical and laboratory data were reviewed retrospectively from medical records.

**Results:**

Among the 28 patients, six patients underwent newborn screening, of which only one was missed. Therefore, 23 patients were diagnosed because of disease onset. Among all the patients, 24 showed varying degrees of symptoms such as rash, vomiting, seizures, and drowsiness, while only four cases remained asymptomatic nowadays. The concentration of 3-hydroxyisovalerylcarnitine (C5-OH) in blood and pyruvate, 3-hydroxypropionate, methylcitric acid, 3-hydroxyvaleric acid, 3-methylcrotonylglycine in urine were increased greatly among affected individuals. After prompt supplement of biotin, both the clinical and biochemical symptoms were dramatically resolved and nearly all patients developed normal intelligence and physique on follow-up. DNA sequencing revealed 12 known and 6 novel variants in the *HLCS* gene of patients. Among them, the variant of c.1522C > T was the most common.

**Conclusions:**

Our findings expanded the spectrum of phenotypes and genotypes for HLCS deficiency in Chinese populations and suggested that with timely biotin therapy, patients with HLCS deficiency showed low mortality and optimistic prognosis. Newborn screening is crucial for early diagnosis, treatment, and long-term outcomes.

**Supplementary Information:**

The online version contains supplementary material available at 10.1186/s13023-023-02656-y.

## Introduction

Holocarboxylase synthetase (HLCS) deficiency (OMIM:253,270) is a rare autosomal recessive disorder of biotin metabolism resulting in multiple carboxylase deficiency (MCD). The worldwide overall incidence of HLCS deficiency has been reported at 1/200,000 live births [1], while the incidence in China is approximately 1/930,600 [2]. In Japan, this disorder occurs more frequently with an incidence of 1/100,000 [3]. HLCS is an enzyme that could catalyze biotin incorporation into carboxylases, including propionyl-CoA carboxylase (PCC), 3-methylcrotonyl-CoA carboxylase (MCC), pyruvate carboxylase (PC) and acetyl-CoA carboxylase (ACC) [4, 5]. The *HLCS* gene*,* located on chromosome 21q22.1, spans approximately 240 kb and comprises 14 exons [6]. A defect in HLCS causes low activities of these biotin-dependent carboxylases, which would subsequently lead to imbalances in the metabolism of amino acids, carbohydrates, and fatty acids.

HLCS deficiency was first known as early-onset MCD because most patients present first clinical manifestations in the neonatal period or early infancy with a variety of symptoms, including skin lesions, vomiting, hypotonia, drowsiness, seizure, metabolic acidosis, hyperammonemia, and developmental delay [4, 7, 8]. If untreated, some affected individuals would progress to severe metabolic acidosis that results in coma or death. Early diagnosis and prompt therapy of biotin supplementation could prevent neurological sequelae and clinical events. For this reason, HLCS deficiency meets the criteria for inclusion in newborn screening programs. Many countries currently perform newborn screening for HLCS deficiency based on tandem mass spectroscopy analysis of elevated 3-hydroxyisovalerylcarnitine (C5-OH) on dried blood spots. The measurement of urine organic acids may reflect the increased level of lactic acid, pyruvate, 3-hydroxypropionate, methylcitric acid, 3-hydroxyvaleric acid, and 3-methylcrotonylglycine consistent with decreased activities of the above carboxylases [1].

In the present study, we studied the clinical, biochemical, and genetic features of 28 patients with HLCS deficiency. The aim of this research was to summarize the current knowledge of the gene variant spectrum, corresponding clinical manifestations, and long-term outcomes in Chinese patients with HLCS deficiency.

## Methods

### Patients

A total of 28 patients with HLCS deficiency who were hospitalized in the Department of Pediatric Endocrinology and Genetic Metabolism, Shanghai Xinhua Hospital, and Newborn Screening Center from 2006 to 2021 were enrolled. General data were collected, including clinical manifestations at onset and symptoms during long-term follow-up. HLCS deficiency was diagnosed by the analysis of biochemical metabolites including increased levels of blood C5-OH and urine lactic acid, pyruvate, 3-hydroxypropionate, methylcitric acid, 3-hydroxyvaleric acid, 3-methylcrotonylglycine, and by molecular analysis of the *HLCS* gene. Parents or legal guardians of the participants signed an informed consent form, approving analysis of their clinical records, and publication of the anonymous data, in agreement with institutional and national legislation. The research was approved by the Ethics Committee of Xinhua Hospital (approval number XHEC-D-2022-063).

### Detection of metabolites

The blood levels of acylcarnitines, including C5-OH, propionylcarnitine (C3), and acetylcarnitine (C2) were detected by tandem mass spectrometry (MS/MS; Applied Biosystems, API 4000, California, United State) on dried blood spots [9]. The ratios of C3/C2 and C5-OH/C3 were calculated at the same time. The levels of urinary organic acids including pyruvate, 3-hydroxypropionate, methylcitric acid, 3-hydroxyvaleric acid, and 3-methylcrotonylglycine were measured by gas chromatography-mass spectrometry (GC/MS; Shimadzu Limited, QP2010, Kyoto, Japan) [10].

### Molecular analysis of the *HLCS* gene

Genomic DNA was extracted from peripheral blood using the DNA extraction kit (TIANGEN Biotech, Beijing, China) according to the manufacturer’s instructions. All 14 coding exons and flanking regions of *HLCS* were amplified using polymerase chain reaction (PCR) as previously described [11], and these PCR products were sequenced on an ABI3700 sequencer (Applied Biosystems, Foster City, California, United State) after purification. Then the ClinVar database, the HGMD database, and the previous literatures were used to identify whether the mutations had been reported. The pathogenicity of novel variants was evaluated based on the American College of Medical Genetics and Genomics (ACMG) standards and guidelines. For novel missense variants, the potential pathogenicity was predicted by Mutation Taster, PolyPhen-2, PROVEAN, and Sorting Intolerant From Tolerant (SIFT), and Amino acid conservation was analyzed by phyloP and phastCons.

### Treatment

Treatment including oral biotin and diet adjustment was commenced immediately after confirming the diagnosis. For patients during acute decompensation, the initial therapy included a low-protein diet, supplementation of biotin as well as L-carnitine, and intravenous fluid therapy with glucose as well as electrolytes. Then the treatment was adjusted according to their response to biotin and other personal condition. The recommended dosage of lifelong administered biotin was 10–40 mg/day [12].

### Statistical analyses

Data that did not significantly deviate from normal distribution were tested using an unpaired two-tailed t-test, and non-normally distributed data were tested using the Mann–Whitney U test. Statistical analyses were performed using GraphPad Prism 5 software (GraphPad Software Inc., San Diego, CA, United States).

## Results

### Clinical characteristics

In this study, a total of 28 patients (17 males, 11 females) were recruited with a confirmed diagnosis of HLCS deficiency. Among them, six patients underwent the MS/MS-based newborn screening using blood C5OH as a biomarker. However, only five cases (5/28, 17.9%) were identified, and one of them showed first clinical manifestations at the age of 19 days due to refusal to retest after receiving the first positive results. The remaining four infants remained asymptomatic after biotin therapy. Among these patients, only one (P24) was missed by newborn screening and diagnosed clinically due to the onset of symptoms, such as scaling erythroderma around the periorificial face, neck, axillae, trunk, and diaper area, respiratory distress, and lactic acidosis. Therefore, a majority of patients (23/28, 82.1%) were diagnosed by selective metabolic investigation using MS/MS due to disease onset with typical symptoms.

The age at onset of 24 patients was shown in Table 1. A majority of affected children suffered first metabolic acidosis before the age of 1 year, and the median onset age was 5 months (range 7 days–19 months). Overall, two-thirds of patients manifested with varying degrees of erythematous rashes on the skin. Among them, only one patient (P27) was characterized mainly by mild cutaneous signs as recurrent eczema on the cheeks and received the treatment of topical corticosteroid as well as a dairy-free diet but without any clinical improvement. Then selective metabolic investigation using MS/MS as well as GC/MS led to the diagnosis of HLCS deficiency. As soon as the start of biotin treatment, her skin lesions recovered greatly. The most common clinical symptoms of these patients were skin lesions (66.7%, 16/24), vomiting (62.5%, 15/24), diarrhea (37.5%, 9/24), respiratory distress (29.2%, 7/24), drowsiness (29.2%, 7/24), coma (20.8%, 5/24), and seizure (12.5%, 3/24). Neurological symptoms including drowsiness, coma, and seizures were also common clinical manifestations in affected ones, which usually appeared in early-onset patients, indicating their clinical symptoms were more serious. In addition, seizures were the initial symptom of the disorder in three patients (P1, P2 and P5), which were particularly sensitive to biotin therapy, often stopping within minutes to hours of administration. A total of 5 patients presented with abnormal images by MRI brain scans, which demonstrated cerebral or cortical atrophy, decreased white matter, or ventricular enlargement.

**Table 1:** Clinical characteristics and mutations of patients with HLCS deficiency

Patient no	Sex	NBS	Onset age	Current health condition	Mutation 1	Mutation 2
1	F	No	1 m	Healthy	c.1994G > C, p.R665P	c.1994G > C, p.R665P
2	M	No	3 m	Healthy	c.1088 T > A, p.V363D	c.1088 T > A, p.V363D
3	M	No	19 m	Healthy	c.1522C > T, p.R508W	c.1522C > T, p.R508W
4	F	No	4 m	Healthy	c.126G > T, p.E42D	c.1088 T > A, p.V363D
5	F	No	5 m	Healthy	c.126G > T, p.E42D	c.1088 T > A, p.V363D
6	M	No	7 m	Healthy	c.1088 T > A, p.V363D	c.1522C > T, p.R508W
7	M	No	8 m	Healthy	c.1522C > T, p.R508W	c.1522C > T, p.R508W
8	M	No	7 m	Healthy	c.1522C > T, p.R508W	c.1522C > T, p.R508W
9	M	No	3 m	Deceased	c.1522C > T, p.R508W	c.1522C > T, p.R508W
10	F	No	6 m	Healthy	c.1522C > T, p.R508W	c.1088 T > A, p.V363D
11	M	No	5 m	Lost to follow-up	c.1522C > T, p.R508W	c.1522C > T, p.R508W
12	M	No	2 m	Deceased	c.1544G > A, p.S515N	c.1481G > T, p.G494V
13	M	No	3 m	Healthy	c.1433C > T, p.T478M	c.1825C > T, p.P609S
14	F	No	2 m	Healthy	c.663_664delCA, splicing	c.1648G > A, p.V550M
15	F	No	2 m	Healthy	c.1544G > A, p.S515N	c.1810G > A, p.V604M
16	F	Yes	-	Healthy	c.1397G > T, p.G466V	c.1522C > T, p.R508W
17	M	No	7d	Healthy	c.780delG,p.G261Vfs*20	c.1522C > T, p.R508W
18	M	No	5 m	Lost to follow-up	c.1522C > T, p.R508W	c.1522C > T,p.R508W
19	M	No	4 m	Healthy	c.1810G > A, p.V604M	c.1522C > T, p.R508W
20	M	Yes	-	Healthy	c.1544G > A, p.S515N	c.1522C > T, p.R508W
21	F	Yes	19d	Healthy	c.780delG,p.G261Vfs*20	c.1522C > T, p.R508W
22	F	Yes	-	Healthy	c.223C > T, p.Q75X	c.1544G > A, p.S515N
23	M	Yes	-	Healthy	c.2057 T > G, p.I686S	c.1985G > A, p.S662N
24	M	Yes	24d	Healthy	c.1825C > T, p.P609S	c.780delG,p.G261Vfs*20
25	F	No	3 m	Healthy	c.1544G > A, p.S515N	c.2126C > T, p.P709L
26	M	No	5 m	Deceased	c.1522C > T, p.R508W	c.1522C > T, p.R508W
27	M	No	7 m	Healthy	c.1522C > T, p.R508W	c.1522C > T, p.R508W
28	F	No	3 m	Healthy	c.1522C > T, p.R508W	c.1088 T > A, p.V363D

M, male; F, female; d, days; m, months; NBS, newborn screening

### Biochemical results at HLCS diagnosis and after treatment

As is shown in Table 2, all of the patients in our study had a level of C5OH far beyond the cutoff, while the blood C3 level and C3/C2 ratio were slightly increased in almost half of the affected individuals. Moreover, the elevated ratio of C5OH/ C3 was found in a large panel of patients. Similarly, abnormal results of urine organic acid analysis were reported for nearly all patients as an elevation of pyruvate, 3-hydroxypropionate, methylcitric acid, 3-hydroxyvaleric acid, and 3-methylcrotonylglycine, suggesting HLCS deficiency. After treatment, all of the biochemical markers above were decreased remarkably, except the blood C5OH of 8 patients, and urinary methylcitric acid as well as 3-hydroxyvaleric acid of 4 patients were beyond the normal range. Additionally, comparing biochemical data before and after treatment, there was a significantly statistical difference (*P* < 0.05).

**Table 2:** Comparison of biochemical data in blood and urine before and after treatment

	C5-OH (μmol/L)	C3 (μmol/L)	C3/C2	C5-OH/C3	Pyruvate (mmol/mol Cr))	3-hydroxypropionate (mmol/mol Cr)	3-hydroxyvaleric acid (mmol/mol Cr))	3-methylcrotonylglycine (mmol/mol Cr))	Methylcitric acid (mmol/mol Cr))
Before treatment (Median, range)	7.01 (3.26–15.01)	4.34 (0.61–14)	0.15 (0.03–0.63)	1.82 (0.12–11.59)	42.05 (4.52–990)	16.21 (4.07–470.3)	212.8 (7.5–550.4)	39.7 (8.54–177)	3.94 (0.9–42.24)
After treatment (Median, range)	0.35 (0.08–1.87)	1.64 (0.62–2.98)	0.08 (0.02–0.11)	0.27 (0.08–4.97)	2.32 (0.0–11.7)	0 (0.0–3.5)	1.1 (0.0–43.32)	1.01 (0.0–3.7)	0.17 (0.0–1.6)
P value	< 0.001	0.007	0.007	0.028	0.013	0.025	< 0.001	< 0.001	0.004
Reference range	0.06–0.6	0.5–4.0	0.0–0.2	0.04–0.25	0.0–30.0	0.0–4.0	0.0–4.0	0.0–5.0	0.0–0.7

### Mutation analysis of the *HLCS* gene

Genetic analysis was performed on all patients. The results of the genetic analysis are shown in Additional file 1: Table S1. Homozygous mutations of the *HLCS* gene were found in 10 patients (35.7%). Overall, 17 different mutations were identified, including 14 missense mutations that accounted for 89.3% (50/56) of alleles; 1 nonsense mutation, and 2 deletions. Among them, the mutation c.1522C > T (p.R508W) occurred with the highest frequency, accounting for 41.1% (23/56). Six novel mutations were detected in *HLCS* gene, including c.663_664delCA, c.1825C > T (p.P609S), c.1397G > T (p.G466V), c.1433C > T (p.T478M), c.2057 T > G (p.I686S) and c.1481G > T (p.G494V). The bioinformatic characteristics of the five novel missense variants (P609S, G466V, T478M, I686S, and G494V) are shown in Table 3.

**Table 3:** Prediction of the potential pathogenic effect of novel missense variants of *HLCS* gene

Mutation	Domain	PROVEAN^a^	PolyPhen-2^b^	SIFT^c^	Mutation taster^d^	Conservation
I686S	C-terminal of BPL	Deleterious	Probably damaging	Damaging	142	Conserved
P609S	catalytic domain	Deleterious	Probably damaging	Damaging	74	Conserved
G466V	catalytic domain	Deleterious	Probably damaging	Damaging	109	Conserved
T478M	catalytic domain	Deleterious	Probably damaging	Damaging	81	Conserved
G494V	catalytic domain	Deleterious	Probably damaging	Damaging	109	Conserved

Biotin protein ligase, (BPL)

^a^PROVEAN prediction: default threshold is − 2.5, that is variants with a score equal to or below − 2.5 are considered “deleterious”, whereas variants with a score above  − 2.5 are considered “neutral”

^b^PolyPhen-2 prediction: probably damaging with a score above 0.909, in contrast, possibly damaging with a score between 0.447 and 0.908, benign with a score under 0.446

^c^SIFT prediction: amino acids with probabilities < 0.05 are predicted to be deleterious, whereas variants with a score above 0.05 are considered “neutral”

^d^Mutation taster prediction: scores range from 0.0 to 215. The more they score, the more deleterious protein mutations

### Clinical outcomes

During 15 years of follow-up, the overall prognosis was optimistic. As for the current health condition by December 2021, a total of 23 (82.1%) cases developed healthy, three (10.7%) cases died, and two (7.1%) cases were lost to follow-up. Among three deceased patients, the age at death was nearly all during the initial phase of severe metabolic crisis without recognition of the disease and timely supplementation of biotin. And all of them presented with symptoms of vomiting, lethargy and coma, which progressed to death. Of the 23 survivors, due to prompt supplementation of biotin, all showed metabolic stability, and their neuropsychomotor development has been age-appropriate.

## Discussion

HLCS deficiency, characterized by variable and non-specific symptoms, usually presents in the first three months of life. Skin manifestations in HLCS deficiency are typically described as scaly, erythrodermic, seborrhea-like, or ichthyosiform [13, 14]. While, in our study, the rash features of patients usually manifested as desquamative skin fold dermatitis, periorificial and intertriginous dermatitis. The patient (P24) from our study was missed on newborn screening, then ascertained by selective metabolic investigation using MS/MS due to severe acidosis, conversely, skin rash was the only feature in the patient (P27). These patients illustrated differences in the severity of presentation and for recurrent, unexplained rash, even in the absence of other clinical findings, HLCS deficiency should be considered. Since cutaneous manifestations are commonly observed in patients with HLCS deficiency, there is a need for dermatologists to be familiar with diverse skin manifestations associated with HLCS deficiency [15].

As MS/MS-based newborn screening has been increasingly used in China, affected infants can be detected before the disease onset. This is beneficial to prevent life-threatening complications of metabolic acidosis, seizure, and hyperammonemia that could lead to irreversible neurological damage and developmental disability, by allowing treatment to be started ideally before onset. Almost all infants detected by newborn screening, due to the prompt supplementation of biotin, did not present any clinical manifestations related to the onset of HLCS deficiency, which illustrated newborn screening is the prerequisite for ensuring early diagnosis and good prognosis of HLCS deficiency [1, 15].

Importantly, the remarkable alterations of activities in biotin-dependent enzymes, such as propionyl-CoA carboxylase and 3-methylcrotony-CoA could be observed in the entire metabolites panel by the MS/MS-based newborn screening. In particular, the moderately elevated C3 level may suggest propionyl-CoA carboxylase deficiency. Similarly, the increased level of C5OH may be related to 3-methylcrotony-CoA carboxylase activity. However, it was worth noting that among a small number of affected infants, the blood C5OH level in NBS might not be above the cutoff, resulting in false-negative reports and delay of diagnosis [16]. Previous studies have already described cases of neonates diagnosed with HLCS deficiency, who simultaneously presented alteration of blood C5OH level and elevation of urinary levels of 3-hydroxyisovalerate, 3-methylcrotonylglycine, lactate and pyruvate [14, 17, 18]. Here, we report, for the first time, some slight alterations in the C3 level, the C3/C2 ratio as well as the C5OH/C3 ratio with exception of C5OH merely at MS/MS testing. These metabolic alterations at MS/MS testing required GC/MS tests for the qualification of 3-hydroxypropionate and methylcitric acid, as direct products of propionyl-CoA carboxylase activity, which is beneficial for differential diagnosis of 3-MCC deficiency and HLCS deficiency. Meanwhile, it may be prudent to adjust the dosage of oral biotin supplementation based on the level of metabolites both in the blood and urine to ensure that the dosing used is indeed sufficient [19].

As previous studies reported, patients with HLCS deficiency have responded to a variable degree to treatment with pharmacologic doses of biotin, which might be related to the genotype [4, 14]. For example, if the variants in *HLCS* gene are located in the C-terminal biotin binding domain or its adjacent region (amino acids 448th–701th), it is deemed as responsive to biotin. Furthermore, if mutations of two alleles are both in the C-terminal biotin binding domain, the biotin dose can even be as low as 1.2 mg/day. In contrast, if the mutation occurs in the N-terminal extension region like the substrate binding region (amino acids 159th–314th), it would lead to a reduction in the affinity of the enzyme for the substrate and show no response to biotin. Therefore, for the patient who has compound heterozygous mutations in the N- and C-terminal regions respectively, the recommended dose of biotin is 20–40 mg/day and for patients who have both allelic mutations located in the N-terminal region, a higher dose may be effective[4]. In patients described here, a dose of 40 mg/day of biotin was given in the initial stage. With close observation on follow-up, it could be possible to decrease the biotin dosage to 10 mg/day for a few patients who had disease onset after the neonatal period due to a reasonable remaining amount of enzymatic activity. We found that this low level of biotin also improved their symptoms and it was able to stabilize the metabolic state and maintain normal growth and development, whereas there were two patients who complied with medication poorly after improvement of symptoms. Fortunately, due to carrying the variant of c.126G > T, which was predicted to be benign, both of them still maintained healthy during our follow-up. However, it has been reported in the literature that if not treated, patients could develop severe clinical manifestations, especially triggered by stressful events, like severe infections. It is precisely for this reason, and considering no side effects of biotin supplementation at commonly used doses, that there is currently a consensus on starting oral biotin supplementation with a dosage ranging between 10 and 40 mg per day. Nonetheless, there are still no precise guidelines on the proper dosage of oral biotin supplementation worldwide.

Consistent with previous studies, the mutations of c.1522C > T and c.1088 T > A might be the hot-spot genes in Chinese populations with HLCS deficiency [11, 20]. In fifteen patients with at least one allele of c.1522C > T, which also included 8 patients homozygous for this mutation, all displayed symptoms after the age of 3 months. A considerable number of them are biotin responsive and the prognosis is fine except in 2 homozygous cases, who died of delay in treatment. This missense mutation has been found in different ethnic groups and belongs to different haplotypes, which suggests a recurrent mutation mechanism [14]. The experiment in vitro confirmed that the c.1522C > T mutation had a higher residual enzyme activity than other mutations and had a better response to biotin supplementation, since this mutation is located in the C-terminal region and generates an elevated Km value [21]. At the same biotin concentration, the residual enzyme activity caused by the c.1088 T > A mutation was mildly responsive to biotin therapy [6], therefore the patient homozygous for this mutation also presented with mild symptoms, such as vomiting and erythematous dermatitis localized in the face, neck, and buttock at the age of 8 months. Noteworthy, in our list of patients, P17, P21 and P24 experienced the onset of disease as neonates, with a median age of 19 days, a much younger age of onset than others. This might be the reason that the mutation of c.780delG severely affected the enzyme activity [6, 22]. However, there is still a disparity between the genotype and biochemical phenotype of HLCS deficiency. In addition, six novel mutations were novel, including one deletion and five missense mutations. The PhastCons values of all novel missense mutations are closer to 1 and the PhyloP scores are all positive, thus these amino sites are being highly conserved. For mutants I686S and P609S, the mutant serine residue is more hydrophilic than the wild-type residues. This loss of hydrophobic interactions might affect the function of the protein, which also occurs in the mutant T478M. And both mutants G466V and G494V lead to glycine into a valine change, in which the mutant valine residue is larger, thereby having a steric hindrance effect. Since the mutant I686S is located at the C-terminal biotin-binding domain, it might result in a lower affinity for biotin. And the other mutants P609S, G466V, T478M, and G494V may also affect the function of catalysis of this domain and are predicted to be damaging.

In conclusion, we described clinical symptoms, biochemical features, long-term outcomes and molecular analysis of 28 patients with HLCS deficiency. The major clinical manifestations of patients with HLCS deficiency are skin lesions and metabolic acidosis. After prompt biotin treatment, most of the affected individuals showed low morbidity, and an optimistic prognosis. Moreover, our findings demonstrated that c.1522C > T is the most common mutation in the Chinese population and the expansion of newborn screening can promote early diagnosis and improve clinical outcomes.

## Supplementary Information

**Additional file 1.** The mutations of *HLCS* identified in the study.

## Data Availability

All data generated or analyzed during this study are included in the article, further inquiries can be directed to the corresponding author.

## Abbreviations

GC/MS: Gas chromatography-mass spectrometry
MS/ MS: Tandem mass spectrometry
HLCS: Holocarboxylase synthetase
PCC: Propionyl-CoA carboxylase
MCC: 3-Methylcrotonyl-CoA carboxylase
PC: Pyruvate carboxylase
ACC: Acetyl-CoA carboxylase
MCD: Multiple carboxylase deficiency
C5-OH: 3-Hydroxyisovalerylcarnitine
C3: Propionylcarnitine
C2: Acetylcarnitine
MRI: Magnetic resonance imaging
