# Supplementary material for: Clinical, biochemical, and genetic analysis of 28 Chinese patients with holocarboxylase synthetase deficiency
Source: Orphanet J Rare Dis. 2023 Mar 8;18:48. doi: 10.1186/s13023-023-02656-y (PMC9997024; doi:10.1186/s13023-023-02656-y)
Supplement: Supplementary file 1 — Additional file 1. The mutations of HLCS identified in the study. [file 13023_2023_2656_MOESM1_ESM.docx]

Supplementary Table 1 List of mutations identified in the current study

| HLCS mutation | gnomAD total frequency | ACMG criteria | Evidence List | References |
| --- | --- | --- | --- | --- |
| c.1994G>C  (p.R665P) | NA | VUS | PM2_P+PM3_P | Wang (2009) Zhonghua Yi Xue Yi Chuan Xue Za Zhi 26，504 |
| c.1088T>A  (p.V363D) | 0.00001591 | P | PS3+PM2_P+PM3_S+PP3 | Dupuis (1999) Mol Genet Metab 66, 80 |
| c.1522C>T  (p.R508W) | 0.00002785 | P | PS3+PM2_P+PM3_S+PP3 | Dupuis (1996) Hum Mol Genet 5, 1011 |
| c.126G>T  (p.E42D) | 0.02359 | B | BA1 | Wang (2009) Zhonghua Yi Xue Yi Chuan Xue Za Zhi 26，504 |
| c.1544G>A  (p.S515N) | 0.00001061 | LP | PM2_P+PM3_S+PP3 | Zheng (2017) Lin Chuang Er Ke Za Zhi 35, 605 |
| c.1481G>T  (p.G494V) | NA | VUS | PM2_P+PP3 | This study |
| c.1433C>T  (p.T478M) | 0.00001061 | VUS | PM2_P+PP3 | This study |
| c.1825C>T  (p.P609S) | 0.00003187 | VUS | PM2_P+PP3 | This study |
| c.663_664delCA | NA | P | PVS1+PM2_P+PM3_P | Luo (2018) J Pediatr Endocrinol Metab 31, 927 |
| c.1648G>A  (p.V550M) | 0.000003976 | P | PS3+PM2_P+PM3_S+PP3 | Dupuis (1996) Hum Mol Genet 5, 1011 |
| c.1810G>A  (p.V604M) | NA | VUS | PM2_P+PP3 | NA |
| c.1397G>T  (p.G466V) | NA | VUS | PM2_P+PP3 | This study |
| c.780delG  (p.G261Vfs*20) | 0.0000177 | P | PVS1+PM2_P+PM3_S | Yang (2000) J Hum Genet 45, 358 |
| c.223C>T  (p.Q75X) | NA | LP | PVS1+PM2_P | Adhikari (2020) Nat Med 26, 1392 |
| c.2057T>G  (p.I686S) | NA | VUS | PM2_P | This study |
| c.1985G>A  (p.S662N) | 0.00001061 | LP | PM2_P+PM3_S+PP3 | Zheng (2017) Lin Chuang Er Ke Za Zhi 35, 605 |
| c.2126C>T  (p.P709L) | NA | VUS | PM2_P | Reid (2016) Brain 139, 2844 |

NCBI RefSeq: all mutations above are base on NM_000411.8; NA, not available; P, pathogenic; LP, likely pathogenic; VUS, uncertain significance; B, benign;
